# Alloreactive cytotoxic T lymphocyte immunotherapy treatment of a patient with metastatic prostate cancer: A case report

**DOI:** 10.1097/MD.0000000000011111

**Published:** 2018-06-15

**Authors:** Junfeng Shi, Yi Chen, Yuetong Chen, Yunzhu Shen, Huanyu Zhao, Hui Sun, Jinfei Chen

**Affiliations:** a Department of Oncology, Nanjing First Hospital, Nanjing Medical University, Nanjing; b Clinical Research Center, Xuyi People's Hospital, Xuyi; c Department of Oncology, Nanjing Pukou Central Hospital, Nanjing; d Jiangsu Key Laboratory of Cancer Biomarkers, Prevention and Treatment, Collaborative Innovation Center for Cancer Personalized Medicine, Nanjing Medical University, Nanjing, Jiangsu Province, People's Republic of China.

**Keywords:** alloreactive CTL, bone metastasis, immune therapy, prostate cancer, PSA/FPSA

## Abstract

**Rationale:**

Cytotoxic T lymphocyte (CTL) immunotherapy is an autologous cellular immune therapy that has been approved for treating patients with malignant tumors. However, there is still limited information regarding the impact of CTL on metastatic prostate cancer (PC) patients with bone metastatic lesions.

**Patient concerns:**

An 82-year-old male patient complained of interrupted urination, urination pain, and significant dysuria on November 24, 2014. Transurethral resection of the prostate (TURP) and postoperative pathological examination showed prostatic adenocarcinoma, and a SPECT/CT scan demonstrated multiple bone metastases. In addition, prostate specific antigen (PSA) and free PSA (FPSA) levels were 54.54 μg/mL and 2.63 μg/mL, respectively, at the beginning of treatment.

**Diagnoses:**

The man was diagnosed with prostatic adenocarcinoma and multiple bone metastases.

**Interventions:**

The patient received 30 cycles of alloreactive CTL (ACTL) immunotherapy regularly.

**Outcomes:**

Over the course of the 2-year treatment, the PC patient exhibited diminished bone metastasis accompanied by a marked reduction of serum PSA and FPSA from 54.54 and 2.63 μg/ml to 0.003 and <0.006 μg/ml, respectively.

**Lessons:**

Our clinical observations demonstrate that CTL immunotherapy is a viable treatment option for PC patients, particularly those with bone metastatic lesions and high serum levels of PSA and FPSA.

## 1 Introduction

Prostate cancer (PC) remains the most common male cancer and the sixth leading cause of cancer-associated deaths in men worldwide.^[1]^ The current treatment options for this disease include surgical removal, endocrine therapy, radiotherapy, and chemotherapy. However, for advanced PC patients with metastatic disease at the time of diagnosis, these treatments appear to have a limited effect. Thus, there is a strong aspiration for developing alternative treatments, including immunity-related or target-based therapy, for use in conjunction with standard treatments.^[2,3]^

Alloreactive CTL (ACTL) immunotherapy is a promising alternative therapy for PC. This therapy is based on the antitumor effect of a unique form of adoptive T cells. These T cells are initially generated by transferring sensitized peripheral blood mononuclear cells (PBMC) from a healthy donor to a tumor-bearing patient.^[4,5]^ According to Hickey et al, the combination of ACTL-based cellular therapy with prodrug activator gene therapy is highly effective against breast cancer with strong brain metastatic potential.^[6]^ Similarly, immunotherapy has shown great promise in the treatment of PC patients.^[7–9]^ In addition, the generation of robust cytotoxic T lymphocytes (CTLs) against prostate-specific antigen (PSA) through priming dendritic cells (DC) with recombinant adenoassociated virus (AAV) has been explored as an adjuvant immunotherapy for the treatment of PC patients.^[10]^ To our knowledge, however, there has been little information regarding the efficacy of ACTL immunotherapy for patients with bone metastasis.^[11,12]^

Here, we describe and discuss a case that uses ACTL immunotherapy for the treatment of a patient with advanced PC. The patient was highly reactive to this therapy, despite being initially diagnosed with bone metastasis and high levels of PSA/FPSA. The steady regression of the disease over the course of the treatment highlights the value of the ACTL immunotherapy for overcoming aggressive PC malignancy in the clinic. The treatment was approved by the Institutional Review Board of Nanjing Medical University, and the patient signed an informed consent.

## 2 Case presentation

**Figure 1:**
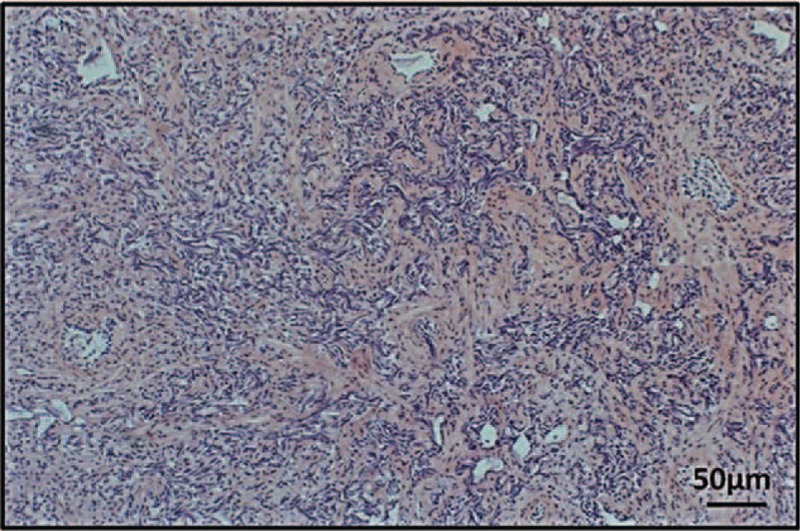
The H&E analysis of postoperative prostate tumors.

**Figure 2:**
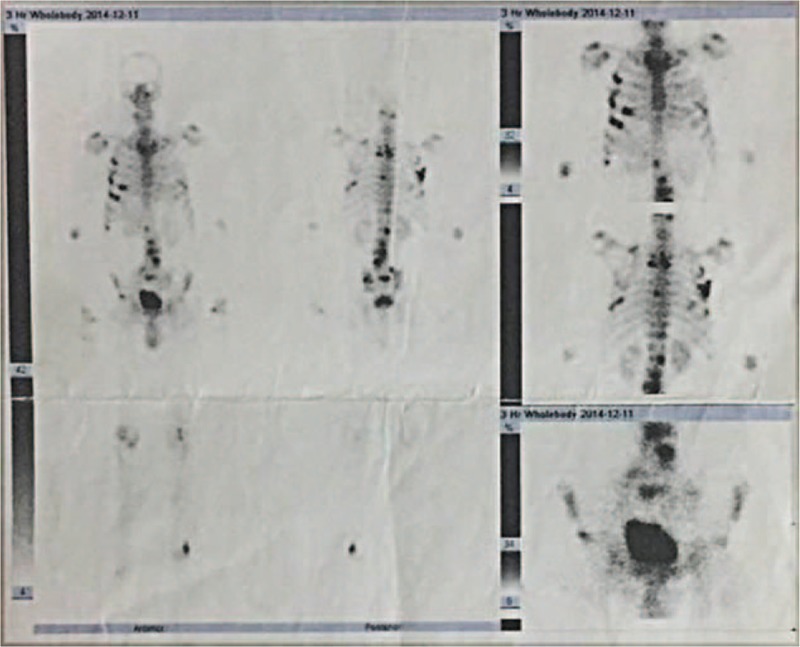
SPECT/CT scans show multiple bone metastases.

**Figure 3:**
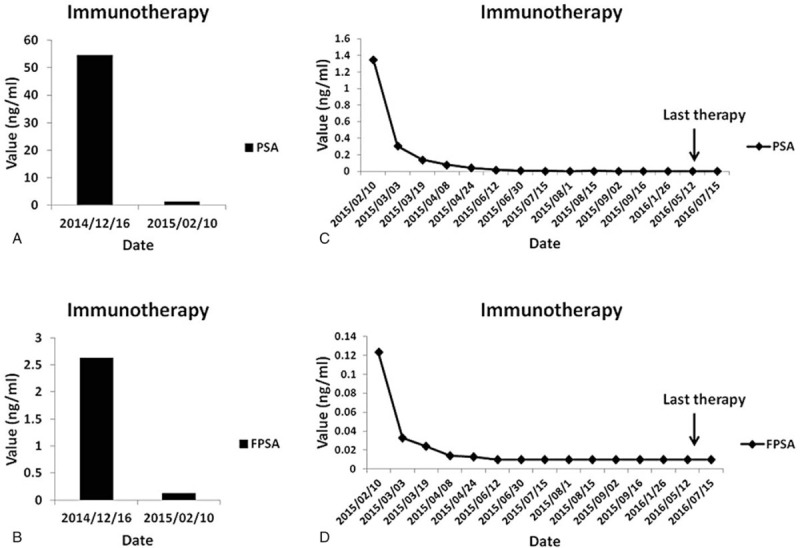
PSA (A and C) and PFSA (B and D) levels during the course of therapy.

An 82-year-old male patient was originally admitted to the hospital for difficulty in urinating spontaneously on November 24, 2014. The patient was diagnosed with prostatic adenocarcinoma after transurethral resection of the prostate (TURP) and postoperative pathological examination (Fig. 1). In addition, a SPECT/CT scan revealed multiple bone metastases (Fig. 2). Initial PSA and free PSA (FPSA) levels were 54.54 and 2.63 μg/mL, respectively (Fig. 3A). The patient exhibited poor tolerance to radiotherapy and chemotherapy, possibly due to advanced age or pacemaker implantation; therefore, immune therapy of alloreactive CTL was selected as a treatment option.

**Figure 4:**
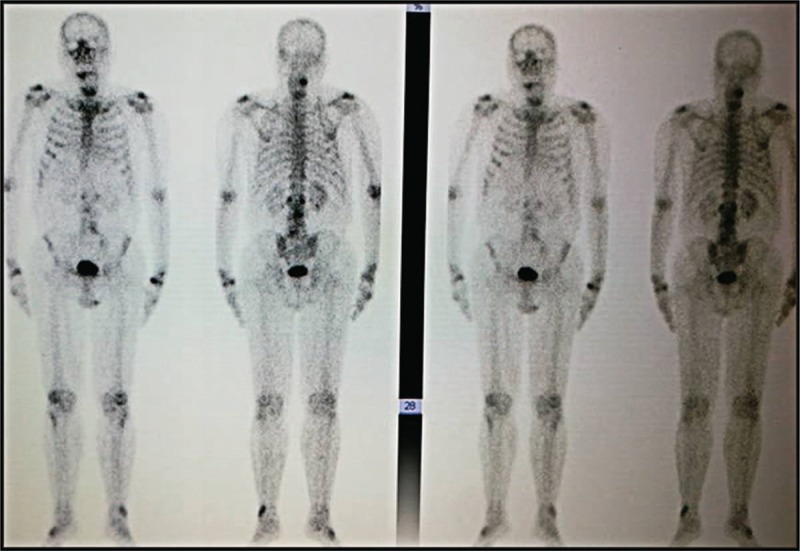
SPECT/CT scans show the disappearance of bone metastases.

**Figure 5:**
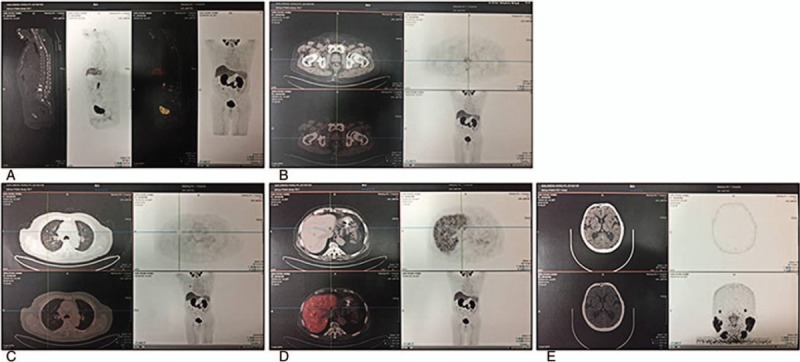
PET-CT scans reflect body (A), prostate (B), lung (C), liver (D), and brain (E) cancer-free metastasis.

**Figure 6:**
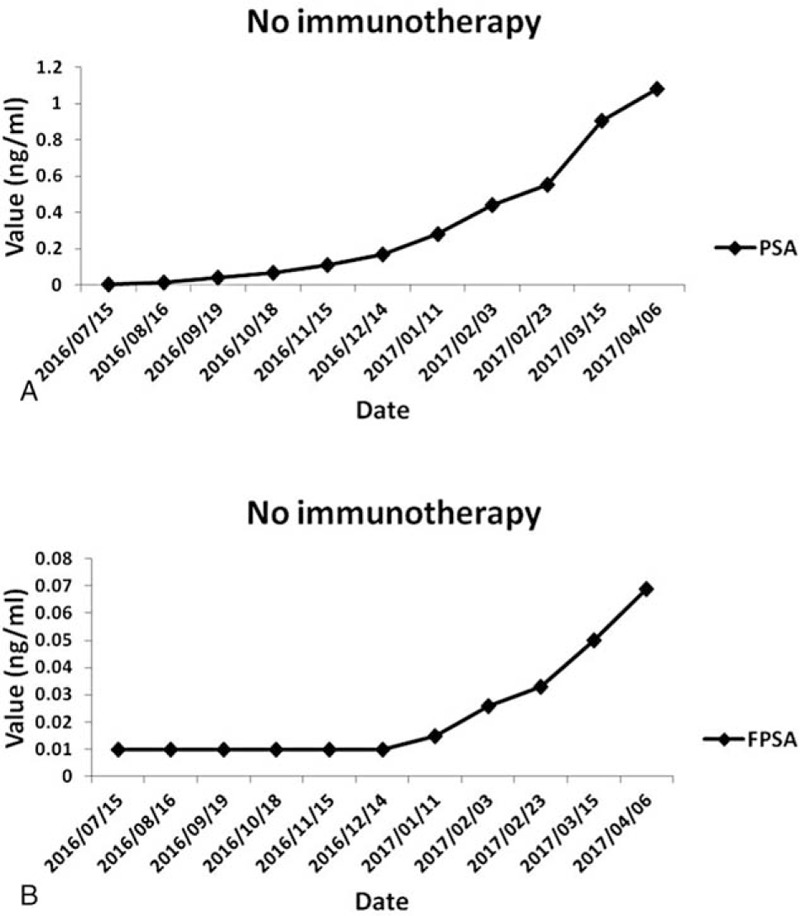
PSA (A) and PFSA (B) levels in the patient.

The patient received the first two cycles of ACTL treatment from December 16, 2014 to February 10, 2015. His PSA levels significantly decreased from 54.54 to 1.35 μg/mL after 28 cycles of continuous ACTL immunotherapy, to undetectable levels of <0.003 μg/mL on May 12, 2016; similarly, immediately after completion of ACTL therapy the FPSA levels were down from 2.63 μg/mL to undetectable levels of <0.01 μg/mL. The PSA and FPSA level remained stable in subsequent days. All the changes in PSA and FPSA are plotted in Fig. 3B, and each test was performed in the same laboratory. Importantly, there were few bone metastatic lesions detected by the SPECT/CT scan in December 2016 (Fig. 4). Similarly, no metastatic lesions were detected in other organs, including the lung, liver, and the brain, by the PET-CT scan. Importantly, the patient remains alive after receiving 2 years of immunotherapy, despite being initially diagnosed with metastatic lesions at multiple sites of the body (Fig. 5). Conversely, the withdraw of the ACTL immunotherapy was accompanied by a gradual increase in PSA levels in the patient from being undetectable (<0.003 μg/mL) on May 12, 2016 to 1.08 μg/mL on April 6, 2017 (Fig. 6). Unfortunately, the ACTL immunotherapy was not applied again because of the expensive cost and lack of proper blood supply.

It is worth noting that there was no apparent cardiovascular system-related symptom or graft-versus-host disease (GvHD) detected in the patient over the course of the immunotherapy treatment, despite the patient being diagnosed with high blood pressure and Type II diabetes at the beginning the administering immunotherapy.

## 3 Discussion

Our report describes the effectiveness of ACTL cellular immune therapy for a patient with PC. In theory, ACTL therapy is based on the principle that professional antigen presenting DC, which are readily isolated from peripheral blood precursors, dictate the antigen-specific immune responses in patients.^[13,14]^ Meanwhile, the AAV is highly effective in delivering antigens and cytokines to DC, strengthening the patient's CTL response to viral antigens.^[15,16]^ In the case of our clinical treatment, the PC patient was initially diagnosed with bone metastasis. These metastatic lesions were reduced or diminished after receiving a total of 30 cycles of ACTL immunotherapy. This effect was also accompanied by a concomitant decrease in the amount of PSA and FPSA in the serum, which are independent predictors for bone metastasis. These results also argue that the impact of ACTL immunotherapy on metastatic PC may occur through a nonchemoradiotherapy-dependent pathway.

We are aware that the PC patient in the present study also received bicalutamide and zoledronic acid over the course of administering of the ACTL immunotherapy. In fact, the change in the levels of PSA or FPSA during the treatment is likely dependent of the effect of bicalutamide,^[17]^ an antiandrogen drug. However, the doses of bicalutamide, given only once or twice a month, were insufficient. Thus, administration of bicalutamide should have limited interference on our assessment of the association between the ACTL immunotherapy and metastatic progression of the disease. Meanwhile, there is lack of evidence in the link between zoledronic acid treatment and disruption of metastatic lesions in PC patients.^[18–20]^ This is also consistent with the current literature, where zoledronic acid treatment is associated with changes in the degree of bone metastasis but not a complete inhibition of metastasis.^[21]^ Thus, the inhibition of tumor metastasis in the PC patient, at its best, is likely attributed to the combined treatment of the immunotherapy and bicalutamide.

## 4 Conclusion

Our observation demonstrates a strong effect of ACTL cellular immunotherapy in inhibiting metastatic PCs and expands the therapeutic window of the therapy.

## Acknowledgment

Written consent was taken from the patient for publishing his clinical details and histopathological photomicrographs.

## Author contributions

**Data curation:** Yuetong Chen.

**Methodology:** Yi Chen.

**Resources:** Yunzhu Shen.

**Supervision:** Jinfei Chen.

**Validation:** Huanyu Zhao, Hui Sun.

**Writing – original draft:** Junfeng Shi.
